# Analysis of 454 sequencing error rate, error sources, and artifact recombination for detection of Low-frequency drug resistance mutations in HIV-1 DNA

**DOI:** 10.1186/1742-4690-10-18

**Published:** 2013-02-13

**Authors:** Wei Shao, Valerie F Boltz, Jonathan E Spindler, Mary F Kearney, Frank Maldarelli, John W Mellors, Claudia Stewart, Natalia Volfovsky, Alexander Levitsky, Robert M Stephens, John M Coffin

**Affiliations:** 1 Advanced Biomedical Computing Center, SAIC Frederick, Frederick National Laboratory for Cancer Research, PO Box B, Frederick, MD, USA; 2 HIV Drug Resistance Program, NCI, PO Box B, Frederick, MD, USA; 3 Division of Infectious Diseases, University of Pittsburgh, Pittsburgh, PA, USA; 4 LMT, SAIC Frederick, Frederick National Laboratory for Cancer Research, Frederick, MD, USA; 5 Tufts University, Boston, MA, USA

**Keywords:** 454 pyrosequencing, HIV-1, Error rate, PCR induced recombination

## Abstract

**Background:**

454 sequencing technology is a promising approach for characterizing HIV-1 populations and for identifying low frequency mutations. The utility of 454 technology for determining allele frequencies and linkage associations in HIV infected individuals has not been extensively investigated. We evaluated the performance of 454 sequencing for characterizing HIV populations with defined allele frequencies.

**Results:**

We constructed two HIV-1 RT clones. Clone A was a wild type sequence. Clone B was identical to clone A except it contained 13 introduced drug resistant mutations. The clones were mixed at ratios ranging from 1% to 50% and were amplified by standard PCR conditions and by PCR conditions aimed at reducing PCR-based recombination. The products were sequenced using 454 pyrosequencing. Sequence analysis from standard PCR amplification revealed that 14% of all sequencing reads from a sample with a 50:50 mixture of wild type and mutant DNA were recombinants. The majority of the recombinants were the result of a single crossover event which can happen during PCR when the DNA polymerase terminates synthesis prematurely. The incompletely extended template then competes for primer sites in subsequent rounds of PCR. Although less often, a spectrum of other distinct crossover patterns was also detected. In addition, we observed point mutation errors ranging from 0.01% to 1.0% per base as well as indel (insertion and deletion) errors ranging from 0.02% to nearly 50%. The point errors (single nucleotide substitution errors) were mainly introduced during PCR while indels were the result of pyrosequencing. We then used new PCR conditions designed to reduce PCR-based recombination. Using these new conditions, the frequency of recombination was reduced 27-fold. The new conditions had no effect on point mutation errors. We found that 454 pyrosequencing was capable of identifying minority HIV-1 mutations at frequencies down to 0.1% at some nucleotide positions.

**Conclusion:**

Standard PCR amplification results in a high frequency of PCR-introduced recombination precluding its use for linkage analysis of HIV populations using 454 pyrosequencing. We designed a new PCR protocol that resulted in a much lower recombination frequency and provided a powerful technique for linkage analysis and haplotype determination in HIV-1 populations. Our analyses of 454 sequencing results also demonstrated that at some specific HIV-1 drug resistant sites, mutations can reliably be detected at frequencies down to 0.1%.

## Background

454 sequencing is a rapid, high throughput sequencing technique used to obtain massively-parallel (or ultradeep) numbers of sequences. Multiplexing with the aid of barcoded primers permits substantial numbers of independent samples to be analyzed simultaneously. High throughput sequencing has greatly facilitated genomic and metagenomic studies of a wide variety of organisms and viruses [1,2] including whole genome sequencing and detection of single nucleotide polymorphisms in population-based screens. In general, these applications involve analysis of a genetically uniform sample or mixtures of individual samples from diploid genomes encoding two alleles at specific loci. However, it has also been applied to samples, such as virus populations, with multiple alleles at a single site [3-11]. For example, 454 sequencing may be useful for detecting minority HIV drug resistance mutations which may contribute to virologic failure [12].

Several limitations inherent in the sequencing technology or introduced during an initial PCR step require careful consideration before ultradeep HIV sequencing data from patients can be analyzed [13]. It is well known that PCR amplification can introduce recombination between templates. For example, it was reported that PCR amplification of two distinct HIV-1 tat gene sequences resulted in the formation of recombinant DNA sequences in up to 5.4% of the amplified products. [14]. Other studies have reported >20% [15], and even 37% [16] of recombinant products after PCR amplification. These high rates of *in vitro* recombination represent a substantial limitation for determining linkage and haplotype composition [17,18]. Yet, descriptions of the effects of PCR-based recombination in ultradeep sequencing derived data are limited [19-24]. In addition, point errors introduced during PCR and sequencing also limit its utility [25]. When the goal is to determine the genome sequence of an organism, this inaccuracy can be compensated for by comparing sequencing reads with a reference and removing any sequence with differences below a certain threshold. For example in a study by Gilbert *et al.*[26], a threshold of 98% was used for determining sequence similarity resulting in removing 15% of the sequence reads. An alternative approach is to assemble many overlapping sequences in order to produce a consensus sequence at each position [27]. These approaches, however, are less useful when the goal is to identify minority variants. Efforts have been made to estimate the average and site-specific error rates by pyrosequencing. Hus, *et al.* studied the accuracy and quality of 454 sequencing on the V6 hypervariable region of cloned microbial ribosomal DNA and estimated that the average error rate was 0.49% per base [13]. Rozera *et al.* reported an error rate of 454 sequencing on HIV-1 *env* quasispecies of 0.97% in homopolymeric regions and 0.24% in non-homopolymeric regions [10]. Similarly, Wang *et al.* reported that the sequencing error rate for four HIV plasmid clones was 0.98% for all types of errors. These studies mainly focused on the average error rate detected by 454 sequencing. Variation in error rate across nucleotide positions is uncertain. Determining the error rate at each specific nucleotide position is essential for detecting low frequency mutations at positions conferring HIV drug resistance.

In the present study, we characterized the sensitivity and accuracy of PCR amplification followed by 454 sequencing for detecting HIV-1 drug resistance mutations, determined the sources for point errors and indels, and measured the rate of PCR-based recombination. Furthermore, we modified the PCR conditions to reduce the rate of recombination and improved the ability of this technique to determine linkage between mutations and haplotype composition in HIV-1 populations.

## Results

To investigate error and recombination rates introduced by the PCR and sequencing steps, three 454 sequencing experiments (Runs 1, 2, and 3) were performed on PCR products generated from HIV RT clones that were either WT (Clone A) or contained 13 drug resistance mutations (Clone B). A total of 774,322 sequences was obtained from 17 samples. Surprisingly, we observed that a few mutant sequences were found in those samples that were supposed to be 100% WT (2 sequences in Run1 MID2 (100% wt) and 2 in Run2 MID2 (100% wt, Table 1). Infrequent WT sequences were also found in the 100% mutant samples (1 in Run1 MID3 (100% mutant) and 6 in Run2 MID3 (100% mutant, Table 1). These results could be due to either a low level of cross contamination between clones occurring while generating the panel of mutant to WT mixtures, or cross contamination during primer synthesis, leading to small fractions of primer DNA molecules with the incorrect MID. In any case, the level of such cross contamination was too low – approximately 0.01% - to affect any of our conclusions.

**Table 1:** Detection of recombination during PCR

**Sample**	**Fragment**	**Mixture**	**Number of sequence**					
			**Total sequence**	**Wild type**	**Mutant**	**Recombinant**	**Observed rate of recombinants**	**Corrected rate of recombinants**
Run1								
MID1	1	100% wild type	8503	8483	0	20	0.24%	0.00%
MID2	1	100% wild type	43699	43480	2	217	0.50%	0.00%
MID5	1	10% mutant	15923	12485	2039	1399	8.79%	8.42%
MID7	1	1% mutant	9548	9329	98	121	1.27%	0.90%
MID3	2	100% mutant	24210	1	24127	82	0.34%	0.00%
MID4	2	100% mutant	13466	0	13432	34	0.25%	0.00%
MID10	2	10% mutant	49169	35477	10156	3536	7.19%	6.90%
MID11	2	1% mutant	12070	11681	171	218	1.81%	1.51%
Run2								
MID1	1	100% wild type	48828	48735	0	90	0.18%	0.00%
MID2^a^	1	100% wild type; Clone DNA/no PCR	98785	98670	2	113	0.11%	0.00%
MID5	1	100% mutant	52287	0	51903	384	0.73%	0.00%
MID7	1	50% mutant	58882	18565	31388	8929	15.16%	14.82%
MID9	1	50/50 mix RNA	54744	14151	27648	12945	23.65%	23.30%
MID3	2	100% mutant	60477	6	60297	174	0.29%	0.00%
MID4	2	100% wild type	38970	38705	0	265	0.68%	0.00%
Run 3								
MID11	1	50% mutant	62437	21163	40777	497	0.78%	0.43%
MID12	1	50% mutant	122327	23589	83134	15604	12.00%	11.65%

^a^ Run2MID2: Cloned DNA without PCR amplification.

### Detection of PCR-based recombination

For the purpose of this study, recombinants were defined as sequences that contained both wild type and mutant bases at the specified drug resistant sites in a single sequence. In Run1 and Run2, using standard PCR conditions (Protocol 1 in methods), 454 sequencing detected a high frequency of PCR-introduced recombination. For example, in Run1 MID7 (1% mutant/99% wt), there were 0.9% recombinants and in MID5 (10% mutant/90% wt) there were 8.42% recombinants. Detectable recombinants increased to 14.82% in Run2 MID7 and 23.30% in Run2 MID9 (both 50% mixtures) (Table 1). We recognized that some of the so called recombinants could be the result of point mutations in a pure wild type or a pure mutant molecule, or recombination with low level cross contaminating templates. When we used 100% WT or 100% mutant as controls (Run1 MID1, 2, 3, and 4; Run2 MID1, 2, and 5), the background recombinant frequencies ranged from 0.11% (Run2 MID2, 100% wt) to 0.73% (Run2 MID5, 100% mutant) (Table 1). Average background recombinant frequencies were taken for each run and were subtracted from the experimental values to obtain corrected recombinant percentages (Table 1).

To attempt to reduce the extent of recombination, modifications were made to the PCR conditions, including a higher concentration of each primer, a more processive polymerase lacking 3′ to 5′ exonuclease proofreading activity, longer elongation time, and fewer cycles of amplification (Protocol 2 in methods). By incorporating these modifications, we were able to reduce recombination rates significantly. For example, in Run3 (50% mutant/50% wt) recombinants were 0.43% (MID11, low recombination PCR) compared to 11.65% by standard PCR (MID12, standard PCR) (Table 1). Overall, changing the PCR conditions resulted in a 27-fold reduction in detectable recombinant sequences.

**Figure 1:**
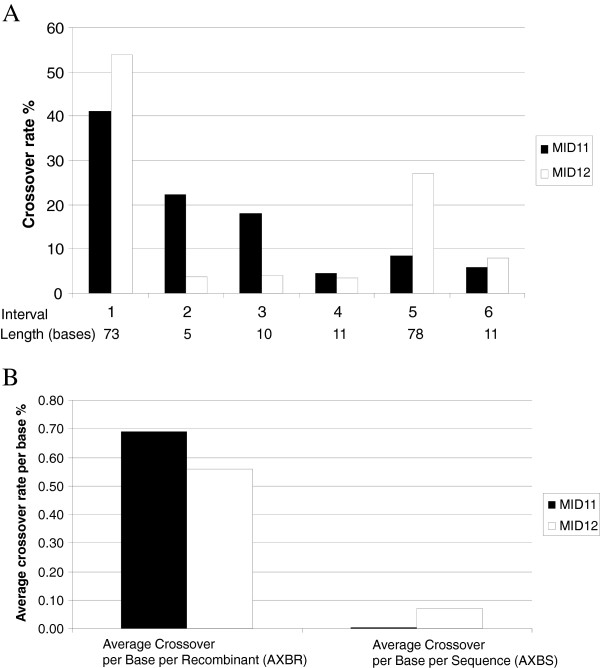
**Recombinant crossover rate estimation. (A)** Observed crossover rates at each interval of recombinant sequences in Run3. The Y axis is the crossover rate in percentage. The X axis shows the interval sites between each drug resistance site and the length of the intervals. **(B)** Normalized average crossover rates. AXBR was obtained by dividing average crossover/base by total number of recombinant sequences. AXBS was obtained by dividing average crossover/base by the total number of all sequences.

We also compared the site-specific crossover frequencies in two samples from Run3. Figure 1A shows the frequency of crossovers in each interval for all the recombinant sequences detected. Generally, the longer the interval between drug resistance sites, the more frequent were the detectable crossovers in that interval. For example, in Run3 MID12 (standard PCR, interval 1 (73 bps), the crossover rate was 54% compared to 3.7% (Chi square test, p < 0.0001) in interval 2 which was only 5 bases in length (Figure 1A). Figure 1B shows the overall crossover frequencies per base for the two PCR conditions. To investigate if different PCR conditions affected the number of crossover events, the average crossover per base per recombinant sequence (AXBR) and the average crossover per base per sequence (AXBS) were calculated. While AXBR was similar in both samples, 0.56% in Run3 MID12 (standard PCR) and 0.69% in Run3 MID11 (low recombination PCR), AXBS was significantly different, 0.07% in Run3 MID12 and 0.006% in Run3 MID11 (Figure 1B). This result indicates that the PCR conditions did not affect the frequency of observed crossover events in a sequence; rather, the low recombination PCR conditions reduced the number of templates involved in recombination. Note that this analysis does not take into account unobserved crossover events involving identical templates.

**Figure 2:**
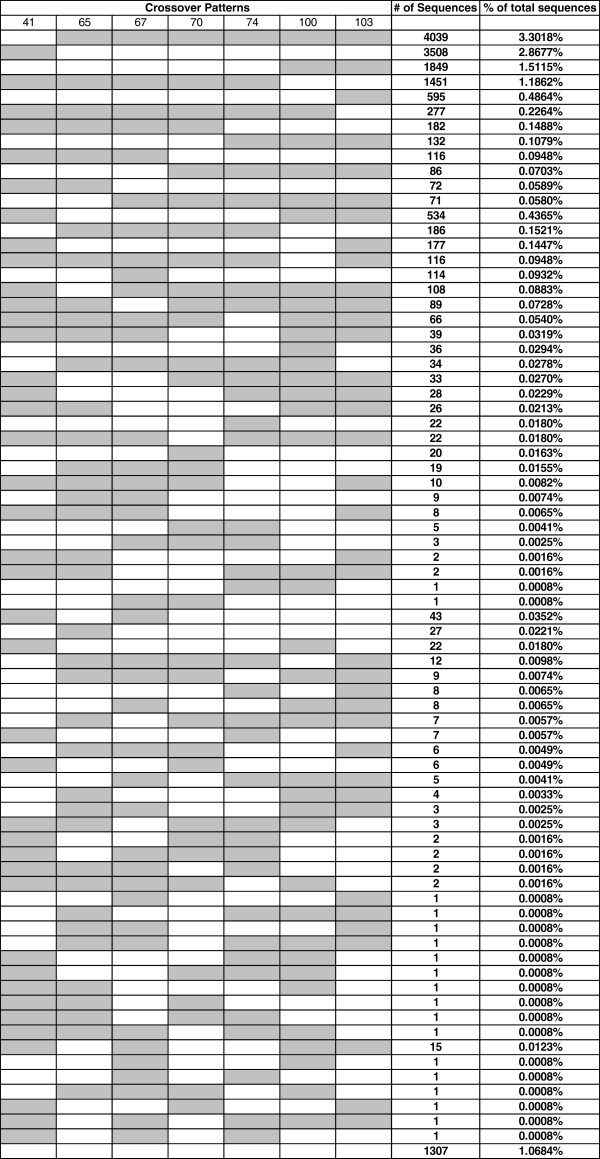
**Crossover patterns of recombinant sequences from Run3 MID12 analyzed using standard PCR conditions.** The second row shows the drug resistance codon positions. The 1st to the 7th columns show the nucleotides at each of the 7 sites with WT marked in white and mutant marked in gray. Detectable crossover events are shown as color changes (from white to gray or from gray to white). The last row represents recombinants that have one or more nucleotides in the drug resistance codons missing from sequencing.

**Figure 3:**
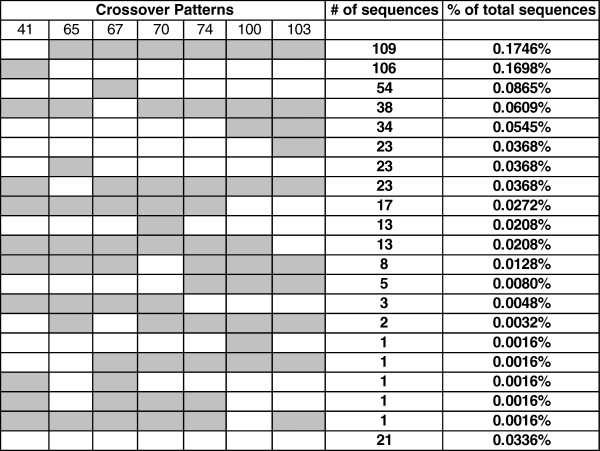
**Crossover patterns of recombinant sequences from Run3 MID11 obtained using low recombination PCR conditions.** The second row shows the drug resistance codon positions. The 1st to the 7th columns show the nucleotides at each of the 7 sites with WT marked in white and mutant marked in gray. Detectable crossover events are shown as color changes (from white to gray or from gray to white). The last row represents recombinants that have one or more nucleotides in the drug resistance codons missing from sequencing.

We used the 7 drug resistance sites as crossover detection signals in the fragment 1 samples. Theoretically, there could be 1024 [11] different patterns because there were 7 intervals within which crossovers could be detected. Seventy-five different crossover patterns (7% of all possible patterns) were found in Run3 MID12 (standard PCR, Table 2), and only 20 detectable patterns were observed (2% of all possible) using the low recombination conditions of Run3 MID11 (low recombination PCR, Table 2). The majority of recombinants were the result of single crossover events. Our results show that it was rare for 4 or more crossovers to occur in a recombinant from Run2 MID12. In fact, there were only 6 recombinants found with 4 crossover events out of 14,297 total recombinants and only a single recombinant found from 5 crossover events in Run3 MID12 (Table 2). In Run3 MID11, there were 3 recombinants from 3 crossover events and no recombinants with 4 or 5 events (Table 2). The detailed crossover patterns between Run3 MID12 and Run3 MID11 are shown in Figures 2 and 3 in which WT nucleotides are marked in white and mutant nucleotides are marked in gray.

**Table 2:** Summary of recombination patterns

	**Run2 MID12 (standard PCR0)**		**Run3 MID11 (low recombination PCR)**	
	**# of patterns**	**# of sequences**	**# of patterns**	**# of sequences**
1 Crossover	12	12378	9	311
2 Crossover	28	1710	8	161
3 Crossover	28	188	3	4
4 Crossover	6	20	0	0
5 Crossover	1	1	0	0
Sum	75	14297	20	476

### Detection of PCR/454 errors

**Figure 4:**
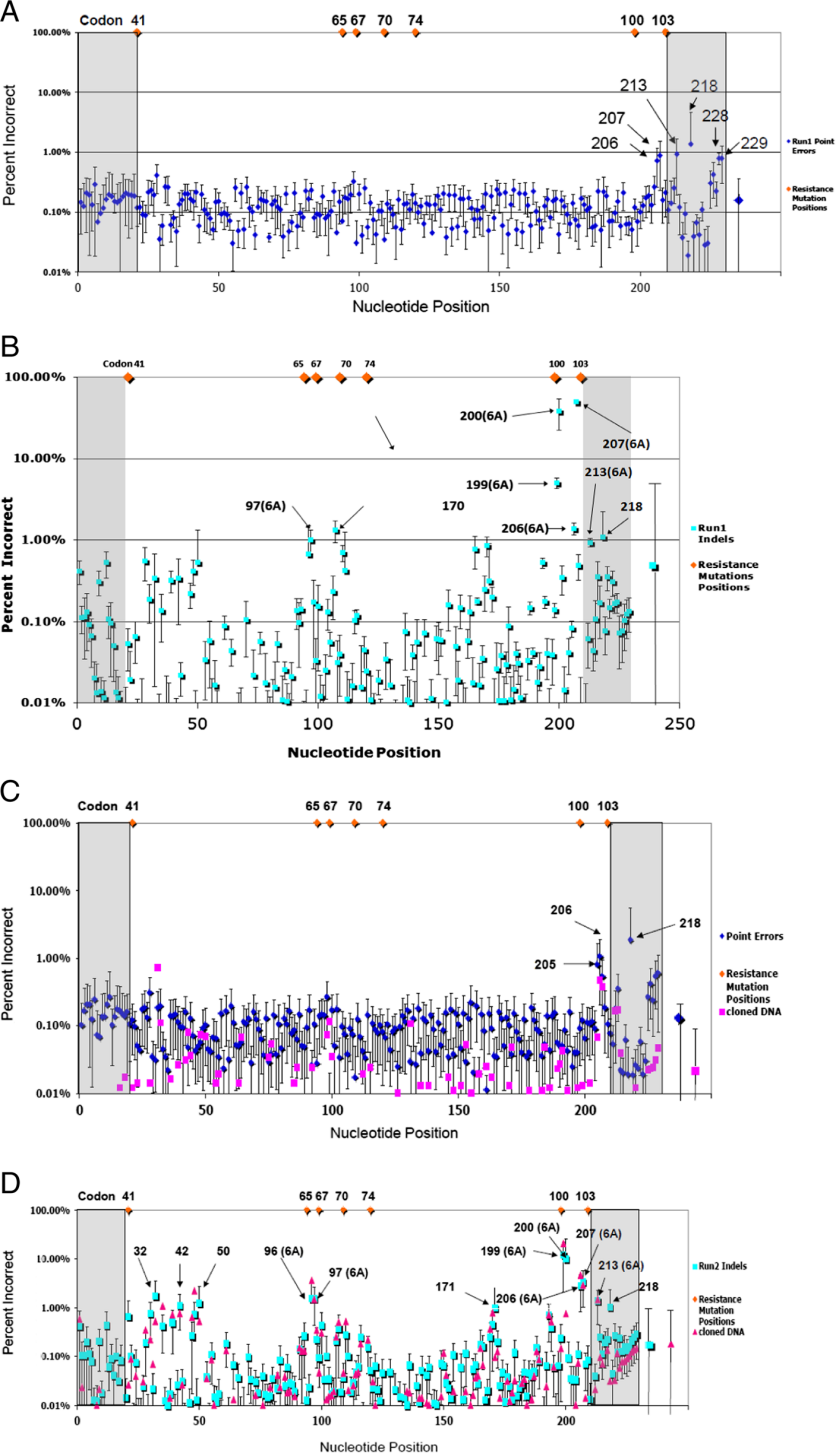
**Point error and indel error estimation. (A)**. Point error distribution in Run1 fragment 1. The data are from Run1 MID1, 2, 5, 7, 8, and 9. The X axis is the nucleotide position of sequences, with position 1 corresponding to nucleotide 101 in BH10 RT [29]. The Y axis is the mean error rate of all samples at each position (blue diamonds). The error bars correspond to 1 standard deviation. The orange diamonds at the top show the locations of drug resistance sites. The numbers with arrows indicate nucleotide positions of sites with high error rates. The gray boxes show the 20 nucleotide PCR primer regions. **(B)** Indel error distribution in Run1 fragment 1, combined MID1, 2, 5, 7, 8, and 9. The Y axis is the mean indel error rate of all samples at each position (blue squares). The error bars correspond to 1 standard deviation of the error rate of each position. Other symbols are as in panel A. The numbers in parentheses indicate the number of As in homopolymer regions. **(C)** Point error distribution in Run2 fragment 1. The data are from Run2 MID1, 5, 7, and 9. Conventions are as in panel A. The pink squares show point errors from cloned sample DNA (MID2). Blue diamonds show the mean error rate of all other samples at each position. **(D)** Indel error distribution from Run2 MID1, 5, 7, and 9. Conventions as in panel B Pink squares show the indel errors of cloned sample DNA. Blue squares are the mean indel error rate of all other samples at each position. The colored symbols and vertical lines after the second gray box show the average errors and standard deviations.

Point mutations and indels can be introduced in both the PCR and sequencing steps. Overall, with samples of either 100% WT or 100% mutants (Run1 MID1-4, Run2 MID1-4, Table 3), we found that that 66.2% of the PCR/454 generated sequences had at least one error, with 56.0% of these sequences having 1 or 2 errors per sequence (Table 3). These errors included both point mutations and indels and varied considerably in frequency from one MID (sample) to another within the same run. To determine whether the error rate was biased relative to one part of the sequence or another, we plotted the point error distribution along the full length of Run1 fragment 1, combining the analyses of MID 1 (100% wt), 2 (100% wt), 5 (10% mutant), 7 (1% mutant), 8 (0.1% mutant), and 9 (0.01% mutant, Figure 4A.) The point errors ranged from 0.02% to 1.36% per base with a mean of 0.15 +/− 0.14% (Table 4). Point errors were distributed evenly along the length of the sequences up to nucleotide position 206. Note that positions 205, 206, 207, and 228 were in homopolymer regions (multiple contiguous identical nucleotides) and positions 228–229 were at the 3′ end of the sequencing reads (Figure 4A). The high error rates at the end of the sequences were due to pair-wise misalignments (see Methods). Figure 4B shows that the indel frequency in Run1 ranged from 0.002% to 49.99% with a mean of 0.51% +/− 4.16% (Table 4). The indels with high frequencies (approximately ≥ 1%) were found in runs of As, which are quite frequent in this fragment. Among those indels, approximately 0.47% were deletions and 0.08% were insertions. Similar trends for point errors (Figure 4C) and indel errors (Figure 4D) were observed in Run2, where the corresponding averages were 0.12 ± 0.16% for point mutations with the amplified samples and 0.25±1.1% for indels with the amplified samples (Table 4). To determine the sources of the errors we cloned a 287 bp amplicon encompassing the fragment 1 region of interest and all primers, keys and MIDs (Run2 MID2, 100% wt, cloned without PCR) necessary for 454 sequencing so that it could be directly sequenced without the PCR step. Figure 4C shows that the point errors of the cloned sample (pink squares) ranged from 0.001% to 0.7% with a mean of 0.02 +/− 0.06% (Table 4) compared to point errors in PCR amplified samples (blue diamonds in Figure 4C), which ranged from 0.008% to 1.06% with a mean of 0.12 +/− 0.16% (Table 4, cloned vs. amplified, with paired *T* test, p < 0.0001). This result shows that the majority (more than 80%) of point errors occurred during PCR amplification. By contrast, there was no difference in indel errors between the cloned and PCR amplified samples, consistent with their generation during the 454 analysis. Indels in the sequences from the cloned sample (pink triangles in Figure 4D) ranged from 0.001% to 20.84% with a mean of 0.25 +/− 1.14% while indels of amplified samples (cyan squares in Figure 4D) ranged from 0.002% to 12.18% with a mean of 0.25 +/− 1.10% (Table 4, cloned vs. amplified, with paired *T* test, p=0.74), indicating that most or all of the indel errors resulted from the 454 sequencing. Overall, among those indels, deletions and insertions were present at approximately the same frequency, 0.18%. We also analyzed the error rates in Run 3 MID11 (low recombination PCR) and in Run 3 MID12 (standard PCR). No significant difference was found (0.08% in Run3 MID11, low recombination PCR vs. 0.11% in Run3 MID12, standard PCR). However, if the error rates are normalized to PCR cycles (45 cycles in Run3 MID11 vs. 25 cycles in Run3 MID12), then the error rate per cycle in low recombination condition was 0.0031% while the error rate per cycle in the standard PCR condition was 0.0025%.

**Table 3:** Distribution of errors per sequence

**Sample (total sequences)**	**0 errors**	**1 errors**	**2 errors**	**3 errors**	**4 errors**	**5 errors**	**6–10 errors**	**>10 errors**	**All sequences with errors**
Run1MID1 (8305)	24.23%	44.32%	18.62%	8.04%	3.05%	1.01%	.067%	0.06%	6293 (75.77%)
Run1MID2 (43699)	25.50%	14.47%	36.56%	15.79%	4.68%	1.53%	1.42%	0.05%	32556 (74.50%)
Run1MID3 (24210)	67.16%	24.51%	5.03%	1.62%	0.85%	0.43%	0.40%	0.02%	7951 (32.84%0
Run1MID4 (13466)	69.69%	22.70%	4.60%	1.45%	0.63%	0.40%	0.52%	0.00%	4081 (30.31%)
Run2MID1 (48825)	23.23%	43.26%	20.37%	7.09%	2.59%	1.27%	1.80%	0.40%	37483 (76.77%)
Run2MID2 (98785)	0.12%	65.26%	23.88%	6.17%	2.33%	0.96%	1.08%	0.20%	95665 (99.88%)
Run2MID3 (60477)	66.18%	23.57%	5.90%	1.96%	0.79%	0.51%	0.98%	0.09%	20451 (33.82%)
Run2MID4 (38970)	60.18%	26.66%	7.64%	2.42%	1.03%	0.70%	1.27%	0.12%	15517 (39.82%)
Number of sequences (336737)	113740	129224	59438	19843	7031	3056	3875	526	222997 (66.22%)
Percentage in all sequences	33.78%	38.38%	17.65%	5.89%	2.09%	0.91%	1.15%	0.16%	

**Table 4:** Summary of point and indel errors arising during PCR and 454 sequencing

	**Point mutation %**				**Indel mutation**			
	**Minimal**	**Maximal**	**Mean**	**Std**	**Minimal**	**Maximal**	**Mean**	**Std**
Run1	0.02	1.36	0.15	0.14	0.002	49.99	0.51	4.16
Run2 amplified	0.008	1.06	0.12	0.16	0.002	12.18	0.25	1.10
Run2 cloned (MID2)	0.001	0.72	0.02	0.06	0.001	20.84	0.25	1.14

We further analyzed the results of these experiments for the nature of the point errors introduced during the PCR and sequencing steps. We found that base specific point errors resulting from transitions (purine to purine or pyrimidine to pyrimidine nucleotide changes) were about 5–10 fold more frequent than those resulting from transversions (pyrimidine to purine nucleotide changes or vice versa) (Table 5). For example in Run1, transitions ranged from 0.04% to 0.10% and transversions ranged from undetectable (0.00%) to 0.03% (Table 5, Run1 fragment1, *T* test P < 0.0001). Our data also show that the specific error rate followed the order of A≥T>G>C (Table 5) with A and T being the nucleotides most susceptible to error. Again, when the analysis was performed on the same DNA fragment without PCR amplification, the error rate was significantly lower. The distribution pattern also is different from the amplified samples (Table 5). For example, transitions were generally more frequent than transversions in amplified samples, but overlapped in the cloned sample (0.01 to 0.06 vs. undetectable (0.00 to 0.01) (Table 5). Additionally, the relative error rates at different bases were different (C>G>A>T) (Table 5). The difference of base-specific error rates between amplified samples vs. the cloned sample could be due to the nature of the DNA polymerase used in PCR. To determine the sensitivity of this approach to detect the drug resistance mutations that can be found in this portion of RT, we assessed the frequency of point errors at drug resistance sites (Table 6). Mutations at these positions ranged from undetectable (0.00%, L90I) to 0.31%, and were relatively lower than that at non-drug resistance sites, largely reflecting the preponderance of transversions in this set of mutations. The error rate differed among the sites examined, implying that mutations at some positions can be detected with greater sensitivity than others (Table 6). For instance, M41L (A to C) could have been detected with a much higher sensitivity (0.02% background error rate, 95% CI 0.01 to 0.03) than K65R (A to G, 0.31% background error rate, 95% CI 0.19 to 0.42). These position specific error rates could be the result of both the particular base at the position and the nucleotide context surrounding the bases.

**Table 5:** **Base-specific error rate **^**a**^

**Correct base**	**Read as:**				
Run1	A	C	G	T	Total
A		0.02%	**0.10%**	0.02%	0.13%
C	0.01%		0.01%	**0.04%**	0.05%
G	**0.09%**	0.01%		0.01%	0.10%
T	0.03%	**0.08%**	0.02%		0.13%
Run2 amplified ^b^	A	C	G	T	Total
A		0.00%	**0.07%**	0.02%	0.09%
C	0.00%		0.00%	**0.03%**	0.03%
G	**0.05%**	0.00%		0.01%	0.06%
T	0.01%	**0.05%**	0.01%		0.07%
Run2 clone (MID2)	A	C	G	T	Total
A		0.00%	**0.04%**	0.01%	0.05%
C	0.01%		0.00%	**0.06%**	0.07%
G	**0.04%**	0.00%		0.01%	0.05%
T	0.01%	**0.01%**	0.00%		0.02%

^a^ Transition errors are marked in bold.

^b^ Run2 amplified samples include MID1 (100% wt), 2 (100% wt, no PCR), 5 (100% mutant), 7 (50% mutant), and 9 (50% mutant); Run2 clone is Run2 MID2.

**Table 6:** Point errors at drug resistance mutation sites

**Position**	**Mutation**	**Run1**	**Run2 **^**b**^	**Highest of 2 run (95 CI)**
21	M41L(A->C)	0.02%	0.01%	0.02% (0.01 to 0.03)
94	**K65R(A->G) **^a^	0.31%	0.09%	0.31(0.19 to 0.42)
99	**D67W(G->A)**	0.07%	0.02%	0.07% (0.01 to 0.13)
109	**K70R(A->G)**	0.09%	0.06%	0.09% (0.03 to 0.16)
120	L74V(T->G)	0.02%	0.00%	0.02% (0.00 to 0.01)
198	L90I(T->A)	0.00%	0.00%	0.00% (0.00 to 0.03)
209	K103N(A->C)	0.02%	0.01%	0.02% (0.01 to 0.03)
210	K103N(A->T)	0.02%	0.02%	0.02% (0.01 to 0.03)

^a^ Transition errors are in bold.

^**b**^ Run1 includes MID1 (100% wt), 2 (100% wt), 5 (10% mutant), 7 (1% mutant); Totally 77,673 sequences analyzed . Run2 includes MID1 (100% wt), 2 (100% wt), 5 (100% mutant), 7 (50% mutant), 9 (50% mutant); Totally 313,523 sequences analyzed.

We also calculated the percentage of drug resistant mutants in the WT: mutant (clone A: clone B) mixtures to further assess the sensitivity of detecting DRM (Table 7). The observed ratios of mutant to WT were not identical but fell within 2 fold of the expected values. For example, in Run1 MID7 (1% mutant), the expected percent of mutant sequences was 1%, and we detected 1.5%. In Run3 MID12 (standard PCR), we expected 50% mutant, and found 74.6% mutant. At each drug resistant site, the mutation frequencies were in general agreement with the observed fraction of mutant reads. For example, in Run1 MID5 (10% mutant), the observed number of reads was 16.9% mutant. The percent mutant at the specific drug resistant site of D67W was 15.42% and at K70R 17.01%. This sample, which contained an average of 16.9% mutant, had a 95% confidence interval of the mean of all seven drug resistant sites of 15.99-17.0%. The same was true for all samples except one. In Run1 MID10 (10% mutant) the number of mutant reads was 22.3%, which was slightly higher than the 95% confidence interval of the mean of all mutant fractions (21.2%) in this sample. Table 7 also shows that in Run1MID7, in which there were 1% expected mutant reads, the percent of mutations at each codon ranged from 1.32% to 1.63%. Considering that the point error rates were about 0.4% for the drug resistance sites overall (Table 6), it is reasonable to estimate the sensitivity for these mutations at 1%. Consequently, mixtures containing 0.1% (Run1 MID8) and 0.01% (Run1 MID9) mutant were not analyzed.

**Table 7:** Drug resistance mutation detection in mixed samples

	**Sequences**		**DRM at each codon %**									
**Sample**	**Expectecd mutant %**	**Observed mutant %**										
Fragment 1 mutations			M41	K65	D67	K70	L74	L160	K103		Mean	95% CI
Run1MID5	10	16.9	16.21	16.94	15.42	17.01	16.79	16.4	16.67		16.49	15.99-17.0
Run1MID7	1	1.50	1.48	1.63	1.32	1.57	1.51	1.45	1.33		1.47	1.37-1.55
Run2MID7	50	61.58	60.26	60.91	60.08	61.53	61.38	61.64	62.24		61.15	60.43-61.87
Run2MID9	50	63.24	61.56	62.60	61.96	630	62.99	62.85	64.10		62.72	61.97-63.48
Run3MID11	50	65.69	65.71	65.67	650	65.69	65.68	65.71	65.72		65.60	65.35-65.84
Run3MID12	50	74.63	74.41	74.48	73.92	74.43	74.32	74.59	75.29		74.49	74.11-74.87
Fragment 2 mutations			Y181	M184	Y188	G190	L205	T215	T215	K219		
Run1MID10	10	22.26	20.35	20.77	21.05	21.07	21.38	21.35	21.37	22.15	21.19	20.75-21.63
Run1MID11	1	2.24	2.18	2.43	2.40	2.21	1.94	1.76	1.73	1.84	2.06	1.83-2.30

Mean: Mean percentage of all drug resistance codons;

All samples were prepared with standard PCR except Run3 MID11 which was prepared with low recombination PCR.

## Discussion

In this study, we evaluated the ability of 454 sequencing of PCR products to accurately portray HIV sequence populations. Using mixtures of cloned DNA containing wild type or mutant sequences at 13 sites associated with resistance to RT inhibitors, we investigated the frequency and mechanisms of point errors, indels, PCR-introduced recombination, and the sensitivity for detecting drug resistance mutations in three independent runs. We looked initially at recombination. We defined a recombinant sequence as one containing both WT and mutant residues generated from mixtures of the two clones. This method is limited by a small background resulting from its inability to determine if a single nucleotide change resulted from a point error or from a (usually double) recombination event in the intervals between drug resistance sites. Furthermore, we were not able to observe recombination between identical parental sequences. To maximize detectable crossover events, we used 50% wt/mutant mixtures. As our result shows (Table 7), the measured wt/mutant ratios were not exactly 50%. This likely reduced the observed recombination. To differentiate whether a mutation in a WT molecule (or a wild type nucleotide in a mutant molecule) is from a point mutation error or from a crossover event, we sequenced clones of 100% WT and 100% mutant samples as controls. Indeed, in experiments in Run1 and Run2, we observed “recombinants” from pure samples at a frequency of 0.11% to 0.73%. Sequences from these samples were not likely to have been recombinants but probably the result of point errors. For our analyses, these small values were subtracted from observed results with mixtures to obtain corrected recombination frequencies (Table 1). Our results show that standard PCR used for sample preparation produced a remarkably high frequency of artifactual recombination. Generally, frequency of crossovers depended on the length of intervals. However, base compositions appeared to affect the crossover rate as well [14]. Tsibris *et al.*[28] reported that *in vitro* recombination was infrequent (0.11% to 0.15%) in a mixture of 3 different clones with the minor species at 1%. We observed 0.9% to 1.5% recombinants in our experiment with 1% mutant clone in a WT background (Table 1), meaning that more than half of the mutant sequences were involved in crossovers.. The different recombination rates in the two studies may be partially explained by different experimental designs (3 clone mixture vs. 2 clone mixture), different parental sequences (HIV-1 *env* V3 loop vs. HIV-1 *pol*), or different PCR conditions. Hedskog *et al.*[21], and Mild, *et al.*[22] reported 0.89% of *in vitro* recombination in their studies. That is lower than the frequency we obtained. The reasons could be [16] the length of the fragment they used to detect crossovers of 14 signature nucleotides (RT amino acid positions 181, 184, 188, 190, 210, 215, and 219) is shorter than ours (positions 41, 65, 67, 70, 74, 100, 103, 181, 184, 188, 190, 215, 219) [29]. We have signature nucleotide from positions 41 to 103. The differences of the intervals and the sequence compositions from position 41 to 181 partially explain the different recombination rates observed. Additionally, Gorzer *et al.*[19] showed that artifactual recombination significantly correlated with the initial amount of DNA used for PCR amplification. Mild *et al.*[22] used 100,000 templates in PCR and observed 0.89% recombinants while we used 1,000,000 templates in our PCR and observed 11.65% recombinants. PCR mediated recombination results from incompletely extended primers annealing to heterologous templates and extending in the next round of elongation [14,16]. By modifying PCR conditions to reduce the probability of premature termination, we found that that PCR mediated recombination could be reduced by 27 fold.

We next examined point and indel errors known to arise during PCR and ultradeep sequencing. It has been previously reported that the error rates differ at homopolymer regions and non-homopolymer regions [10,13,18]. We found that the point errors were evenly distributed except at homopolymer regions, particularly near the 3′ end of the sequenced region. This discrepancy is even more dramatic in indel error distributions. A high frequency of indel errors was found primarily in homopolymer regions (Figures 4B and 4D). Additionally, we found that, overall, in our study of the HIV-1 RT region, that there were more deletions than insertions in our samples. This is different from the observation by Vandenbroucke *et al.*[24]. They reported 0.07%–0.14% insertions and 0.02% to 0.08% deletions in their study of the HIV-1 *env* V3 region. This difference may be related to sequence context [24]. Manual examination of the sequence alignment confirms that more deletions were produced in homopoly A regions, particularly the region near RT K103. Also, we noticed that the deletion/insertion rate was different between Run1 and Run2 while point mutation errors were very similar. This different deletion/insertion rate may reflect variation in performance of 454 sequencing from one run to the next. We also found that transversion errors were 5–10 fold lower than transition errors (Table 5). Huse *et al.* reported that A to G and T to C changes were more frequent than other types of changes [13]. Our results show that the frequencies of transitions exhibited a small bias in the same direction, but that all transitions were nonetheless more frequent than transversions.

Ultradeep sequencing has been used to identify low frequency drug resistance mutations [3,6,11]. Mitsuya *et al.* proposed that it was unlikely that variants at a frequency > 1.0% resulted from sequencing errors. They used 1% as the cut-off for drug resistance sites and 2% for other RT sites [6]. A similar result was obtained by Gilles *et al.*[30]. Based on 100% wild type or 100% mutant samples, we show that it is possible to use a substantially lower cutoff for some drug resistance sites because error rates were considerably lower at some sites than at others. For example, the background of K103N (A to C) and, K103N (A to T) were each 0.02%, and L74V (T to G) was 0.02%, with 95% confidence bounds of 0.01 to 0.03, and 0.00 to 0.03, respectively (Table 6). We measured the fractions of mutations in samples with mixed wild type and mutant sequences (Table 7) and found that frequencies at each site were in good agreement with expected values down to about 1%. It seems clear from these results, however, that it is possible to use this technology to measure the frequency of specific mutations, particularly transversions, down to less than 0.1%, similar to that achievable with allele-specific PCR [31]. In such cases, however, it is essential -- and not particularly difficult -- to include internal controls of cloned DNA (or transcripts prepared from cloned DNA) to assess the actual background frequencies achieved in any experiment.

The sources for point errors in 454 generated sequences were from both the PCR and the sequencing steps. Although errors resulting from sequencing have been reported to result in part from more than one molecule being bound to a single bead before the emulsion PCR [13], this artifact cannot have caused the errors observed in sequencing cloned DNA. In any case, our data show that PCR contributed the majority (0.12+/−0.16% of PCR amplified vs. 0.02+/−0.06% of cloned, DNA Table 4) of the point error rate and that sequencing contributed primarily to indel errors. This conclusion was also suggested by Vandenbroucke *et al.*[24].

We observed 0.01% cross contamination in our studies (Table 1, Run1 MID2 (100% wt), MID3 (100% mutant), and Run2 MID2 (100% wt, cloned without PCR). This effect could have resulted from laboratory error, but could also be due to cross contamination in primer synthesis resulting in mislabeling of a fraction of a sample with an incorrect MID. We have also shown that a high frequency of recombination could be introduced by standard PCR conditions. However by using the low recombination PCR conditions described here, 454 sequencing technology can be a useful tool in studying mutation linkage and haplotype composition. Our results also have shown that, while indel errors were more frequently found in homopolymer regions and occurred mainly during sequencing, point errors were more or less evenly distributed in the whole region, and occurred mainly during PCR. We found that drug resistance sites had lower point error rates compared to other sites, implying that it is possible to detect rare drug resistance mutations with high sensitivity.

In this study, we observed higher than expected mutants/wt ratios (Table 7). The differences between the expected and observed ratios could be due to the fact that a sequence read was defined as a mutant or wild type by BLAST comparing it to the wild type reference and the mutant reference. If it aligned better with wild type reference (with higher E score), then it was defined as wild type. For the purpose of Table 7, we did not separate recombinants as we did for Table 1; all sequences were assigned either to wild type or mutant. Figure 2 shows the recombination patterns in Run3MID12. It shows that the numbers of the crossover product pairs were not exactly the same. There are more sequences with more gray regions (mutations) than the white regions (wild type). Therefore, more putative recombinants in Table 7 were defined as mutants. Ratios of the mixtures were verified by ASP prior to deep sequencing so the higher than expected mutant sequences are likely due to PCR or sequencing bias.

Recently, Jabara *et al.*[32] reported an experiment system in which a randomly synthesized 8 base segment (“primer ID”) was incorporated into the primer for cDNA synthesis. Consensus sequences were built from the products of PCR amplification and used for mutations detection. By consensus sequence construction, minor sequencing errors and recombination produced by PCR can be removed.

## Methods

### Construction of mutant plasmids

Site directed mutagenesis was performed on an HIV-1_BH10_ WT molecular plasmid clone (designated clone A) [29] to generate a multi-drug resistant mutant clone containing the following RT mutations: 41L, 65R, 67N, 70R, 74V, 100I, 103N, 181C, 184V, 188C, 190A, 215Y, 219Q (designated Clone B). The full sequence has been deposited in GenBank under accession number JX198552.

### Preparation of WT and mutant transcripts

An 895 bp PCR product from codon 22 to 291 in RT was amplified from each of the HIV-1_BH10_ WT and mutant plasmids and cloned into pPCR-Script Amp SK (+) transcription vector using the PCR-Script Amp Cloning Kit (Stratagene). Transcripts were made from each clone, using the RiboMax transcription kit (Promega), quantified spectrophotometrically at 260 nm, diluted to 10^8^ copies/μl, divided into aliquots and stored at -80°C.

### Primer design for use with the 454 standard genome Sequencer system

The Roche Genome Sequencer (GS) FLX System provides sequence reads up to approximately 250 bases. In order to investigate two regions of interest in the HIV-1 RT region that included a number of well characterized drug resistance mutations, primers were designed for fragment 1, a 265 base pair (bp) amplicon encoding amino acids 41 thru 103 of RT, and fragment 2, a 160bp amplicon encoding amino acids 181 thru 219. To sequence 12 samples containing the RT region of interest in run 1, 12 forward primers of 49 bp were designed for fragment 1 and consisted of 4 parts: sequencing primer A, (5′GCCTCCCTCGCGCCA3′), a key (TCAG), a 10 bp Multiplex Identifier (MID; also known as a barcode; see Additional file 1: Table S1) to differentiate among samples, and the HIV target-specific region (5′TAGTAGAAATTTGTACAGAA3′). The reverse primer (39bp) for fragment 1 consisted of 3 parts: sequencing primer B, (5′GCCTTGCCAGCCCGC3′), the same key, and the target region (5′TCCAGTACTGTTACTGATTT3′) and was used for all fragment 1 samples. The 12 forward 454 primers for fragment 2 were also 49 bp and consisted of the same sequencing primer A, key, and MID sequences but a different target region (5′ AAAATCCAGACATAGTTATC 3′). The reverse primer for fragment 2 (5′GCCTTGCCAGCCCGCTCAGGGAGGTTCTTTCTGATGTTT3′) was 39 bp and was used for all fragment 2 samples. These same primers were also used for runs 2 and 3.

### 454 Runs and samples

In all, 3 separate 454 runs were performed on 17 samples (Table 1). Among these samples, 9 were either 100% wild type or 100% mutant, serving as controls to detect background point and indel error rate. The rest were mixtures of wild type and mutant and used for measuring recombination and for detecting specific low level drug resistance mutations.

### Preparation of the clone for PCR error control

To differentiate the errors introduced by PCR from the errors introduced by pyrosequencing a bacterially-grown clone was sequenced directly (without PCR). To generate the clone the WT plasmid was amplified with primers 3-1F, 5′GCCTCCCTCGCGCCATCAGACGCTCGACATAGTAGAAATTTGTACAGAA3′ and 4-1R, 5′GCCTTGCCAGCCCGCTCAGACGCTCGACATCCAGTACTGTTACTGATTT3′. The resulting product included the forward and reverse sequencing primers A and B, the key, MID 2 and the HIV target region from fragment 1. This 265 bp piece was cloned into a pPCR-Script Amp SK (+) vector. The clone was transformed into ultracompetent cells, expanded, purified and digested with restriction enzymes to result in a 287 bp piece of bacterially grown DNA encompassing all primers, keys and MIDs necessary for the successful 454 sequencing of fragment 1.

### Preparation of mixtures and PCR conditions

Both the WT and mutant plasmid clones were quantified spectrophotometrically, and mixed at ratios of mutant to WT at 100%, 50%, 10%, 1%, 0.1%, 0.01%, and 0%. To ensure the accuracy of the ratios, mixtures were analyzed by allele specific PCR (ASP; data not shown) [31]. All mixtures resulted in a final copy number of 10^6^ total copies/μl. The plasmid mixtures were amplified in two fragments using the following PCR conditions (Protocol #1): 400 nM each primer, 200 μM dNTPs, 4 mM MgSO_4_, 1X Hi Fi Buffer (Invitrogen) and 2.5 units Hi Fidelity Platinum Taq (Invitrogen). Following a 2 minute thermal activation of the Taq at 95°, 45 cycles of PCR amplification were performed with each cycle consisting of 95° for 30 sec, 50° for 30 sec and 72° for 30 sec.

In addition, the 50:50 mixtures were amplified using a low recombination PCR protocol (Protocol #2) as follows: 1μM each primer, 200μM dNTPs, 2.3mM MgCl_2_, 1X Taq Gold buffer, 5 units Taq Gold (Applied Biosystems). Following a 15 minute thermal activation of the Taq at 95°, 25 cycles of PCR amplification was performed with each cycle consisting of 95° for 15 sec, 51° for 30 sec and 68° for 1 min 30 sec. All final PCR products, as well as the MID 2 clone grown in E. coli, were quantified using the QuantiFluo DNA Assay Kit (BioAssay Systems) and ~10^12^ copies of each sample was sent to be sequenced using the Roche Genome Sequencer (GS) FLX System.

### Preparation of samples from RNA transcripts

WT and mutant RNA transcripts were mixed at a 50:50 ratio to result in final copy numbers of 2×10^5^, 2×10^4^, and 2×10^3^ copies/μl. Each mixture was used as template with the following conditions: 1X reaction mix (Invitrogen SuperScript One-Step RT-PCR kit) and 200nM each primer. The reactions were denatured at 65º for 10 minutes and placed on ice. RT/Platinum Taq enzyme (Invitrogen) (1μl) was added to each reaction and placed under the following thermocycling conditions: 50º for 30 min, 94º for 2 min, 57.5º for 30 sec, and 70º for 40sec for 35 cycles.

### Sequencing error rate estimation

Sequence reads from the 454 sequencing were sorted into different sample sets according to their MID. After removing the 10 base MID, sequences were aligned to the wild type sequence or the mutant sequence using blastn in BLAST program. A Perl script was written to parse the pair-wise alignments. Any sequence shorter than the length of the reference by 20 or more bases was removed from further analysis. Additionally, ambiguous base calls were not used in the analyses. Because blastn will produce deletions at the ends of alignments, if a mutation or indel at or near the end of the read, leading to a truncation of a sequence, a function was constructed in the script to compare the 5′ region with reference sequences and correct errors at the 5′ end to restore the truncated fragments. No effort was taken to correct the misalignment at the 3′ end due to the fact that this portion was in the 3′ primer region which is not amplified during PCR and was not used for mutation or recombinant detection. This procedure therefore produced high error rates at the 3′ ends (Figures 4A and 4C). To estimate the sequencing error rate, sequencing reads were compared with the reference for each site, any nucleotide difference between a sequencing read and the reference cloned DNA used as template was treated as a sequencing error.

### Recombination detection and rate estimation

To estimate recombination rates each sequence was aligned to the WT or mutant reference sequence. If a sequence was more similar (based on a smaller E value produced by BLAST) to the WT reference then it was considered WT; otherwise, it was considered mutant. If a sequence contained a combination of WT and mutant nucleotides at the 13 drug resistance sites, it was counted as recombinant. This approach could overestimate the number of recombinant sequences if mutations were introducing by PCR error. This potential artifact was evaluated using control experiments with 100% wide type samples (Table 1, Run 1 MID1 (100% wild type) MID2 (100% wt); Run 2 MID1 (100% wt) MID2 (100% wt without PCR amplification)) or 100% mutant samples (Table 1, Run1, MID3 (100% mutant), MID4 (100% mutant); Run 2, MID1-5 (from 100% wt to 50% mutant). And the recombinant frequency was adjusted accordingly.

For crossover rate estimation in each interval between the 13 signature nucleotides, the number (N) of sequences with wild type nucleotide at one site and mutant nucleotide at the other site was counted. The crossover rate in an interval was calculated by dividing the number of crossover events in this interval (N) by the total crossover events in all 6 intervals (T) being expressed in percentage (N/T * 100).

### Frequency of drug resistance mutations in mixtures

Sequences were assigned to be WT or mutant based on the E value produced by BLAST and percentages were calculated from those assignments. The number of drug resistant mutations (DRM) at each of the 13 drug resistance sites was calculated the same way as the sequencing error estimate (above) from these mixed samples. The percentage of each DRM was calculated by dividing the number of sequences containing each DRM by the total number of reads (WT and mutant) in a sample minus the number of sequences containing deletions in the DRM site.

### Statistical test

Student’s *T*-test, Chi Square test, and confidence intervals were calculated with a web application Graphpad (http://www.graphpad.com/quickcalcs/index.cfm). All p values are two-tailed.

## Conclusion

Standard PCR amplification results in a high frequency of PCR-introduced recombination precluding its use for linkage analysis of HIV populations using 454 pyrosequencing. We designed a new PCR protocol that resulted in a much lower recombination frequency and provided a powerful technique for linkage analysis and haplotype determination in HIV-1 populations. Our analyses of 454 sequencing results also demonstrated that at some specific HIV-1 drug resistant sites, mutations can reliably be detected at frequencies down to 0.1%.

## Competing interests

The authors declare that they have no competing interests.

## Authors’ contributions

WS analyzed data and wrote the manuscript; VB, JS, CS performed the experiments; NV, AL processed upstream 454 raw data; JC, MK designed the experiments; FM, JM, RS participated the discussion of experiments and manuscript writing; all authors read and approved the final document.

## Supplementary Material

Additional file 1: Table S1 The sequences of MID used in this study. Click here for file
