# Supplementary material for: Analysis of 454 sequencing error rate, error sources, and artifact recombination for detection of Low-frequency drug resistance mutations in HIV-1 DNA
Source: Retrovirology. 2013 Feb 13;10:18. doi: 10.1186/1742-4690-10-18 (PMC3599717; doi:10.1186/1742-4690-10-18)
Supplement: Additional file 1: Table S1 — The sequences of MID used in this study. [file 1742-4690-10-18-S1.doc]

Supplement Table 1. The sequences of MID used in this study
